# Dobutamine-induced alternations in cerebral blood flow of healthy adults: a 3D pseudocontinuous arterial spin labeling study

**DOI:** 10.1186/s12916-023-02928-1

**Published:** 2023-07-03

**Authors:** Tingting Zhang, Haijun Niu, Yawen Liu, Linkun Cai, Dong Liu, Erwei Zhao, Min Li, Wenjuan Liu, Jing Li, PengGang Qiao, Wei Zheng, Pengling Ren, Zhenchang Wang

**Affiliations:** 1 School of Biological Science and Medical Engineering, Beihang University, Beijing, China; 2 Department of Radiology, Beijing Friendship Hospital, Capital Medical University, Beijing, China; 3 National Space Science Center, Chinese Academy of Sciences, Beijing, China; 4 Clinical Epidemiology and EBM Unit, Beijing Friendship Hospital, Capital Medical University, Beijing Clinical Research Institute, Beijing, China

**Keywords:** Arterial spin labeling, Brain perfusion, Cerebral blood flow, Dobutamine stress

## Abstract

**Background:**

It is unclear whether dobutamine, commonly used clinically in echocardiography and short-term congestive heart failure treatment for promoting increased myocardial contractility, affects brain microcirculatory behavior. Cerebral microcirculation plays an important role in ensuring adequate oxygen transport. Therefore, we investigated the effects of dobutamine on cerebral hemodynamics.

**Methods:**

Forty-eight healthy volunteers without cardiovascular or cerebrovascular disease underwent MRI to obtain cerebral blood flow (CBF) maps using 3D pseudocontinuous arterial spin labeling before and during the dobutamine stress test. Additionally, cerebrovascular morphology was obtained based on 3D-time-off-light (3D-TOF) magnetic resonance angiography (MRA). Electrocardiogram, heart rate (HR), respiration rate (RR), blood pressure, and blood oxygen were simultaneously recorded before and during dobutamine injection and during recovery (not during MRI). The anatomic features of the circle of Willis and the basilar artery (BA) diameter were assessed on MRA images by two radiologists with extensive neuroimaging experience. Binary logistic regression was used to test for the independent determinants of CBF changes.

**Results:**

HR, RR, systolic (SBP), and diastolic blood pressure (DBP) significantly increased after dobutamine infusion. Blood oxygen levels remained similar. Compared to the CBF in the resting state, the CBF values exhibited significantly lower CBF levels in both grey matter and white matter. Furthermore, compared with the CBF in the resting state, that in the stress state was decreased in the anterior circulation, mainly in the frontal lobe (voxel level *P* < 0.001, pixel level *P* < 0.05). Logistic regression showed that body mass index (BMI; odds ratio [OR] 5.80, 95% confidence interval [CI] 1.60–21.01, *P* = 0.008], resting SBP (OR 0.64, 95% CI 0.45–0.92, *P* = 0.014), and BA diameter (OR 11.04, 95% CI 1.05–116.53, *P* = 0.046) were significantly associated with frontal lobe CBF changes.

**Conclusions:**

Dobutamine-induced stress significantly decreased CBF in the frontal lobe anterior circulation. Individuals with a high BMI and low SBP during the dobutamine stress test are more likely to have a stress-induced CBF decrease. Thus, attention should be paid to blood pressure, BMI, and cerebrovascular morphology of patients undergoing dobutamine stress echocardiography or those receiving intensive care or anesthesia.

## Background

Dobutamine is a sympathomimetic drug widely used in clinical practice for cardiac stress testing for the noninvasive detection of coronary artery disease; it is also used in intensive care and anesthesia to manipulate cardiovascular parameters [1–3]. Under the action of dobutamine, an increase in cardiac output (CO), heart rate (HR), and blood pressure occurs, with incidences of tachycardia, arrhythmia, and hypertension [4, 5]. Changes in CO, blood pressure, and HR have been reported to affect cerebral blood flow (CBF), which plays a significant role in the metabolism and energy supply of the brain [6–8]. When the CBF is below the critical value, even only for a few seconds, syncope occurs [9]. Clinically, patients undergoing cerebral bypass surgery or who have chronic heart failure are prone to cerebral hypoperfusion, in which dobutamine is used to increase arterial blood pressure to prevent cerebral ischemia [6]. However, it is unknown whether dobutamine administration results in greater cerebral perfusion. Therefore, considerable effort has been devoted to exploring the effects of dobutamine on CBF [6, 10].

Clinically, dobutamine has been suggested to be associated with increased CBF in patients [11]. In contrast, another study reported that dobutamine caused a decrease in CBF in five patients based on transcranial color Doppler imaging (TCDI) [7]. Moreover, a further study using TCDI on ten healthy volunteers showed that dobutamine did not significantly change cerebrovascular homeostasis [12]. In comparison, an animal experimental study in a model of subarachnoid hemorrhage, in which CBF was acquired with continuous arterial spin labeling (ASL), showed that acute cardiac support with dobutamine administration improved hypoperfusion [8]. The contradictory results of these studies reflect the complex mechanisms of action of dobutamine.

To date, the effects of dobutamine on CBF, as well as its influencing factors, have been explored using different techniques and in patients or animals with different diseases. However, it is important to consider the variations between the CBF measurement techniques when interpreting the data. In the earlier studies, CO, blood pressure, and HR were altered using different doses of dobutamine, and CBF was measured using TCDI or MRI. It is possible that methodological heterogeneity may result in inconsistent results, particularly in patients with cerebrovascular diseases [13–15]. Therefore, the effects of dobutamine on CBF in both patients and healthy subjects remain unclear and are probably complex, influenced by CO, blood pressure, and cerebral blood vessel morphology. Moreover, changes in microcirculatory behavior in the brains of healthy subjects after dobutamine infusion may occur even with normal systemic hemodynamics and could contribute to hypoperfusion or hyperperfusion. From a clinical perspective, dobutamine is widely used to increase blood pressure and CO, but these effects may be accompanied by a risk of death, especially when high doses of dobutamine are administered. Therefore, the effect of dobutamine on cerebral hemodynamics should be considered [16].

As a noninvasive imaging technique, ASL perfusion magnetic resonance imaging (MRI) could directly yielding quantitative CBF measurement [17, 18]. Multiple studies have demonstrated that the CBF measurements obtained by the ASL technique and positron emission tomography (PET) are highly correlated [19–21]. Compared with TCDI, ASL is applied to detect CBF quantitatively in the whole brain, even in the microcirculation. For its practical advantages, this technique is an exceptionally powerful tool for investigating subtle brain changes in clinical research studies for diagnosis and treatment assessment [22]. ASL signals can be imaged using a variety of readout sequences, in which the three-dimensional pseudocontinuous ASL (3D-pcASL) sequence provides a higher signal-to-noise ratio [23, 24].

Therefore, we sought to explore CBF changes in healthy subjects after dobutamine infusion using the 3D-pcASL sequence. We hypothesized that dobutamine-induced stress would decrease CBF, which would be associated with vital signs and cerebrovascular morphology of individuals, such as blood pressure, HR, and cerebral artery diameter.

## Methods

### Participants

Fifty-two healthy volunteers were recruited via advertising. The subjects were between 23 and 34 years of age, and all underwent conventional psychological screening. All subjects were nonsmokers with no history of neurological disorders or head injuries. The exclusion criteria were as follows: any ocular disease; any cardiovascular or cerebrovascular diseases; any psychiatric disorders; any systemic diseases that may influence brain blood perfusion, such as hypertension; and MRI ineligibility (e.g., heart valve replacement, or implanted metal devices). Four volunteers were excluded because they had typical contraindications to MRI or dobutamine administration. All volunteers were instructed to refrain from smoking cigarettes and ingesting tea, coffee, β-blockers, and anti-angina medication for at least 24 h before the MRI study.

The experimental protocol was approved by the Research Ethics Committee of Beijing Friendship Hospital, Affiliated with Capital Medical University (2020-P2-155). Before the experiment, all the subjects signed an informed consent form.

### Study design and stress test procedure

**Fig. 1:**
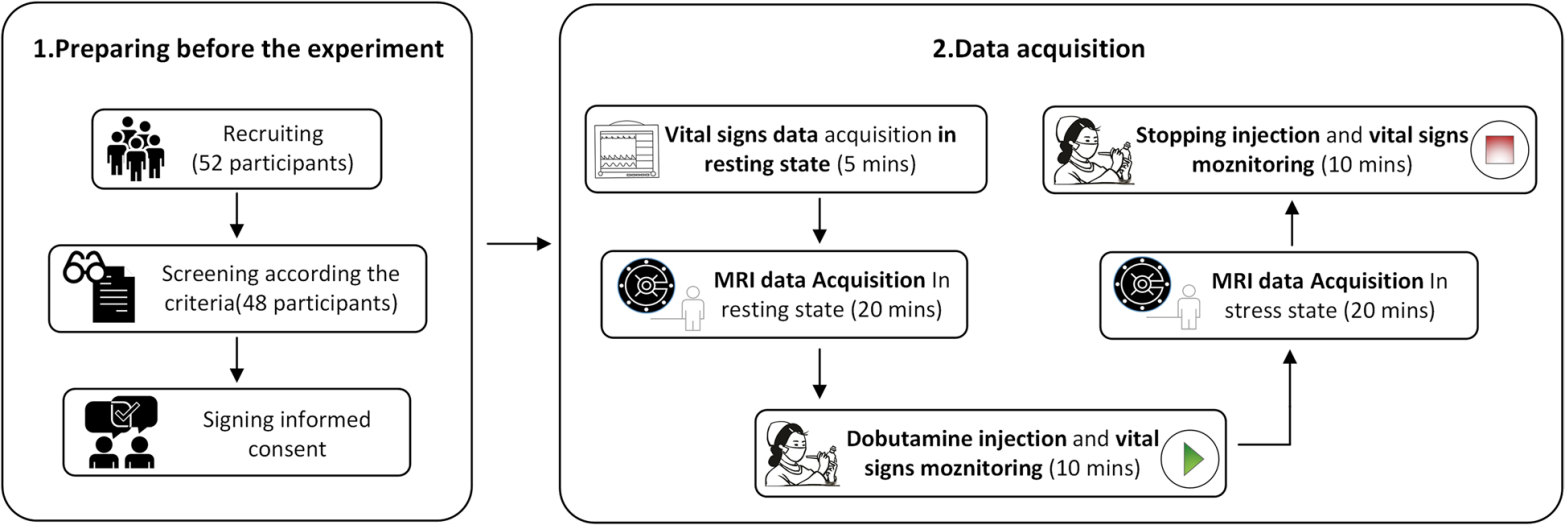
Flowchart of the study

This self-controlled study investigated the effect of dobutamine stress on brain perfusion. Figure 1 shows a flowchart of the experiment. After the acquisition of MRI scans in the resting state, following standard procedures, dobutamine infusion with a starting dose between 5 and 20 μg/kg/min was started. The criteria for reaching the condition for cardiac stress testing were that the systolic blood pressure in the stress state increased by 20% when compared to the resting state or that the heart rate of the volunteer in the resting state was 50% higher than that in the stress state. The injected dose of dobutamine was set lower than the routine clinical method to ensure the safety of the volunteers. During dobutamine infusion, electrocardiogram findings, HR, respiration rate (RR), blood pressure, and blood oxygen of volunteers were recorded using a portable Multi-parameter Vital Signs Monitor (Mindray, BeneVision, Shenzhen, China). Dobutamine administration was maintained until the MRI data was acquired. If there was any discomfort or the systolic blood pressure reached 220 mmHg in the process, the infusion stopped. It has previously been shown that the effect of dobutamine reaches a steady state at 2 min after starting infusion and remains steady during the subsequent continuous infusion of the drug [25]. The responses of most volunteers after dobutamine infusion in this study were consistent with those of a previous study [25]. After the physiological state of the volunteers was stable, MRI scans were obtained in the dobutamine stress-induced state. The MRI scan was conducted only after achieving a steady state defined as a < 5-mmHg change in the mean arterial pressure over 2 min. At the end of the MRI scan, the dobutamine dose was slowly reduced, and physiological data were examined continuously until the HR, blood pressure, and blood oxygen values had stabilized. There were no adverse reactions in the 48 healthy volunteers after dobutamine infusion or throughout the experiment according to the adverse reactions documented in the literature [26]. However, a fast heart rate and respiration rate, occurred at the injection site.

### MRI data acquisition

All participants were examined using a 3.0-T MRI scanner (Ingenia 3.0 T; Philips Healthcare, Best, The Netherlands) with a 16-channel phased-array coil. Foam padding and earplugs were used for each participant to attenuate head motion and scanner noise, respectively. All volunteers underwent high-resolution T1-weighted imaging, a 3D-pcASL scan, 3D-time-off-light (3D-TOF) magnetic resonance angiography (MRA) in the resting state, and a 3D-pcASL scan in the stress state. The parameters of high-resolution T1-weighted imaging were as follow: 200 axial slices, slice thickness = 1.0 mm, repetition time (TR) = 10.3 ms, echo time (TE) = 1154.7 ms, field-of-view (FOV) = 240 mm × 240 mm. The parameters of 3D-pcASL sequence were as follows: 36 axial slices, slice thickness = 4 mm; postlabel delay = 1500 ms; TR/TE = 4852/10.7 ms; reconstruction matrix = 128 × 128; FOV = 240 × 240 mm; number of excitations = 3. 3D-TOF MRA acquisition was performed with 300 slices, TR = 23, TE = 3.5, matrix = 1024 × 1024, FOV = 200, voxel size = 0.5 × 0.5 × 1 mm. Participants were asked to keep their eyes closed and not to think of anything in particular and were instructed to avoid falling asleep.

### Quantitative CBF maps

The CBF images originated from ASL-difference images and proton density-weighted reference images. Data preprocessing was performed using Statistical Parametric Mapping (SPM8) and the Data Processing and Analysis of Brain Imaging (DPABI) toolbox implemented in MATLAB (Version R2014a; MathWorks, Natick, MA, USA). Using a nonlinear transformation from SPM8, we registered CBF images to a PET perfusion template in the Montreal Neurological Institute (MNI) space. The mean co-registered CBF map was defined as the standard CBF template. The CBF maps of all participants were co-registered to the standard CBF template in the MNI space and were resampled to a voxel size of 2 × 2 × 2 mm using the SPM toolbox. Each co-registered CBF image was spatially smoothed with a Gaussian kernel of 6 × 6 × 6 mm full-width at half-maximum to reduce interindividual differences and increase the SNR (signal-to-noise ratio). The whole-brain grey matter (GM) and white matter (WM) were selected as regions of interest (ROIs) for the region-specific analyses. Whole brain GM and WM perfusion in the left and right hemispheres were extracted from the smoothed CBF images using GM and WM templates in MNI space. The CBF maps were then normalized by dividing the value of cerebral blood flow in each voxel with the mean value of the whole brain CBF using the DPABI package.

### Anatomic features of the circle of Willis and Basilar artery

Previous studies have suggested that the contributions of perfusion territory to deep-brain structures vary greatly, which is partly due to the variations in the anatomic features of the circle of Willis [27, 28]. Therefore, the anatomical features of the circle of Willis were assessed. MRA images were analyzed for the presence of vessels in the circle of Willis. According to the criteria given in the literature [29], a vessel was defined as ‘‘normal’’ if the vessel diameter was greater than 1.0 mm; a vessel was defined as hypoplasic if the vessel diameter was less than 1.0 mm.

**Fig. 2:**
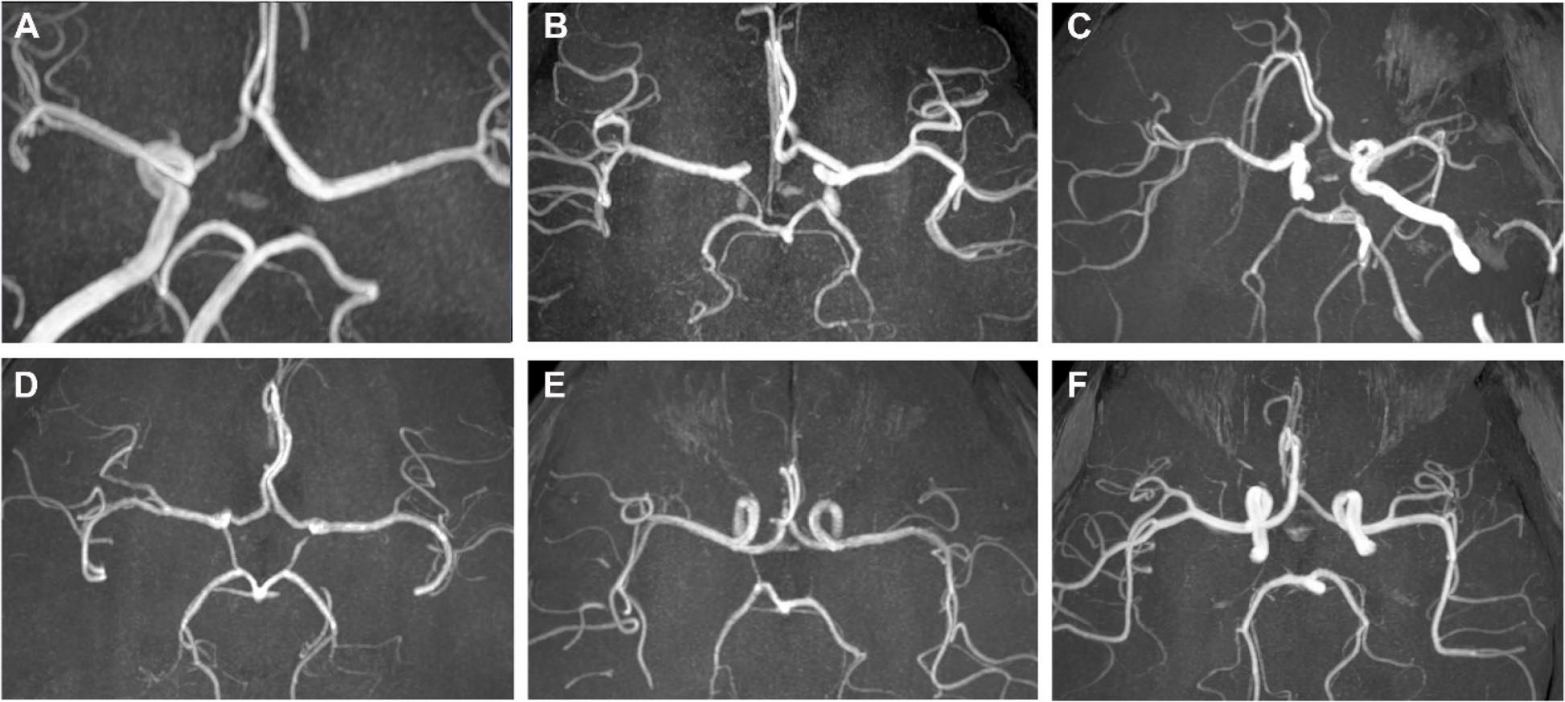
The samples of different types of the circle of Willis on MRA. **A**-**C** show types of the anterior circle of Willis: complete circle of anterior half Willis (**A**, type I), absence or hypoplasia of A1 segments (**B**, type II), absence of the anterior communicating artery (**B**, type III); **D**-**F** show types of the posterior circle of Willis: complete circle of posterior half Willis (**D**, type I), absence of unilateral posterior communicating artery (**E**, type II), absence of the bilateral anterior communicating artery (**F**, type III). MRA, magnetic resonance angiography

The criteria for the types of the circle of Willis were as follows: the typical circle of Willis was divided into anterior and posterior halves, in which the anterior half consisted of the anterior communicating arteries, bilateral A1 segments, and bilateral terminal segments of the internal carotid artery (ICA), while the posterior half consisted of bilateral posterior communicating arteries, bilateral P1 segments, and the basilar artery (BA). Based on the classification method in previous studies and the actual clinical practice of this study, we classified three anterior half-Willis and three posterior half-Willis types (Fig. 2). Figure 2A–C show types of the anterior arc of the circle: a complete circle of anterior half-Willis (Fig. 2A, type I), absence or hypoplasia of A1 segments (Fig. 2B, type II), absence of the anterior communicating artery (Fig. 2C, type III). Figure 2D–F show types of the posterior arc of the circle: a complete circle of posterior half-Willis (Fig. 2D, type I), absence of unilateral posterior communicating artery (Fig. 2E, type II), absence of the bilateral anterior communicating artery (Fig. 2F, type III).

**Fig. 3:**
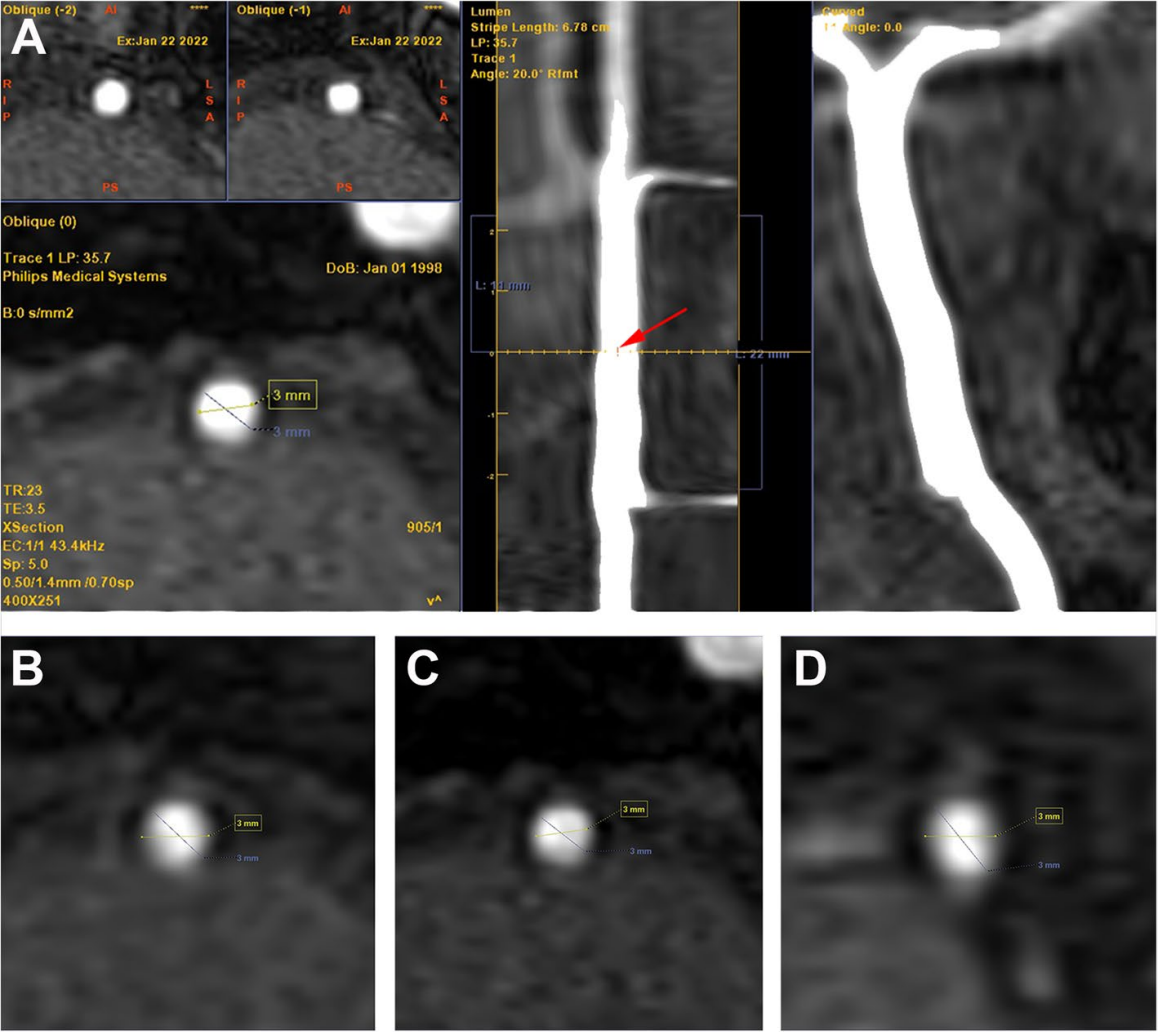
The samples of measurements of basilar artery diameter

In addition, the diameter of the BA at the mid-pons level was measured based on MRA images (Fig. 3). All measurements and evaluations were conducted by two radiologists with extensive experience in neuroimaging and diagnosis. Discrepancies were resolved by discussing and reaching a consensus between the two radiologists.

### Statistical analysis

#### Demographic data

Analyses of demographic data were performed using SPSS 20.0 (Chicago, IL, United States). Wilcoxon’s signed-rank tests were used to calculate differences in clinical characteristics between the two states. Statistical significance was set at a two-tailed probability level of < 0.05.

#### Mean GM and WM CBF

To assess potential group differences in GM CBF and WM CBF, Wilcoxon’s signed-rank tests were performed in SPSS. Moreover, the differences of CBF laterality in GM and WM between the two time points were also examined by using Wilcoxon’s signed-rank tests (the difference of CBF was defined as the CBF values in the left hemisphere minus that in the right hemisphere).

#### Voxel-based analysis

The voxel-based comparison of normalized CBF was conducted using a paired *t*-test to identify CBF variations between the two time points. For multiple comparison correction, we used a false discovery rate [FDR]-corrected method (voxel level, P < 0.001; pixel level, P < 0.05; cluster size, > 719). The results were visualized using the xjView toolbox (xjView 9.5) and the BrainNet Viewer (BrainNet Viewer 1.61). Each cluster showing a significant difference was saved and was used as a mask for subsequent region-of-interest (ROI)-based analyses. The mean CBF values of the ROIs in the 48 subjects were extracted from the smoothed CBF maps using the xjView toolbox.

#### Logistic regression analysis

Binary logistic regression analysis was used to identify predictors of CBF responses to dobutamine-induced stress. The variables considered were age, body mass index (BMI), HR, RR, systolic blood pressure (SBP), diastolic blood pressure (DBP) in the resting state, integrity of the circle of Willis, and BA diameter. The difference in CBF between the two states was introduced as the binary dependent variable; if the difference in CBF was greater than the median of the differences in CBF, the label was set to 1; otherwise it was set to 0. Regression analysis was performed in SPSS 20.0 with the significance level set at *p* < 0.05.

## Results

### Demographics and physiological data

Forty-eight healthy volunteers participated in the study. The descriptive characteristics and physiological data for the rest and stress states are shown in Table 1. HR, RR, SBP, and DBP showed significant increases from baseline to stress state (HR: 68.00 [59.25–75.00] vs 84.50 [70.25–97.50] bpm, *P* < 0.001; RR: 17.00 [15.00–20.00] vs 18.00 [15.00–22.00] bpm, *P* = 0.003; SBP: 117.00 [111.50–127.25] vs 159.50 [151.00–165.00] mmHg, *P* < 0.001; DBP: 73.00 [68.25–80.00] vs 82.50 [78.00–88.00] mmHg, *P* < 0.001). Blood oxygen levels did not differ significantly between the two states (99.00 [98.00–100.00] vs 99.00 [98.00–100.00] %, *P* > 0.05).

**Table 1:** Background of participants

Characteristics	Resting state	Stress state	*P* value
Number of participants (male/female)	48 (43/5)		-
Age, years	25.00 (24.00–28.00)		-
BMI, kg/m^2^	22.67 (21.00–23.67)		-
BMI < 25, %	85.42		
25 ≤ BMI < 30, %	12.50		
BMI ≥ 30, %	2.08		
Heart rate, BPM	68.00 (59.25–75.00)	84.50 (70.25–97.50)	< 0.001
Respiration rate, RPM	17.00 (15.00–20.00)	18.00 (15.00–22.00)	0.003
BA Diameter, mm	3.00 (2.55–3.50)		-
Blood pressure			
SBP, mm Hg	117.00 (111.50–127.25)	159.50 (151.00–165.00)	< 0.001
DBP, mm Hg	73.00 (68.25–80.00)	82.50 (78.00–88.00)	< 0.001
Blood oxygen, %	99.00 (98.00–100.00)	99.00 (98.00–100.00)	> 0.05

Data were presented by median (lower quartile, upper quartile)

*Abbreviations*: *BPM*,Beat per minute, *BA* Basilar artery, *SBP* Systolic blood pressure, *DBP* Diastolic blood pressure

### Differences in CBF maps between the two states

**Fig. 4:**
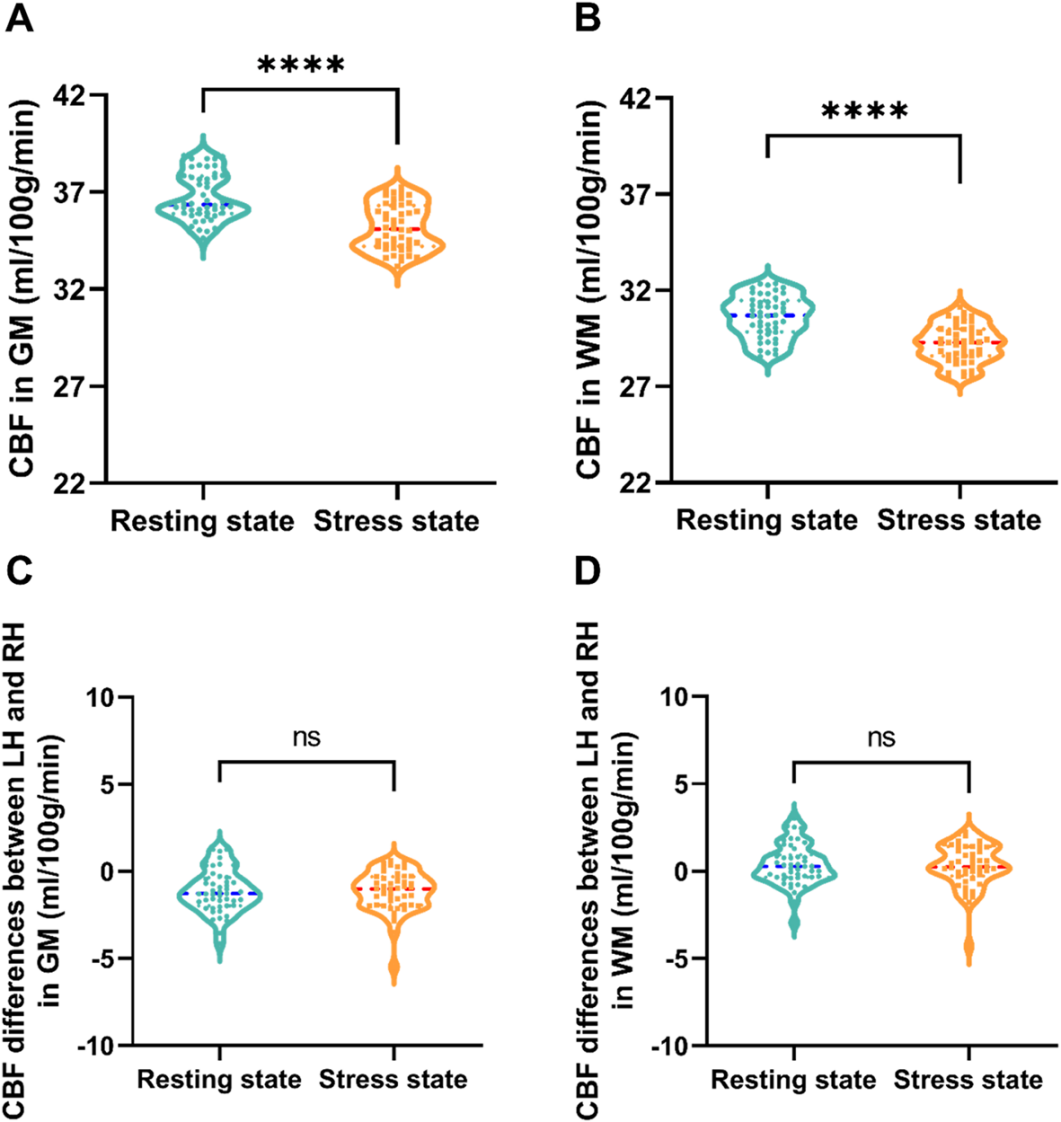
Changes of cerebral blood flow (CBF) in grey matter (GM) and white matter (WM) between the resting state and stress state. **A**-**B** Decreased perfusion in GM and WM after dobutamine infusions compared with the resting state. **C** The difference in GM CBF between the left and right hemispheres in the resting and stress states did not differ significantly. **D** The difference in WM CBF between the left and right hemispheres in the resting and stress states did not reveal any significant differences. ****: *P* < 0.0001; ns: *P* > 0.05

In comparison to the CBF in the resting state, the CBF values exhibited significantly lower CBF levels in both GM and WM (CBF in GM: 36.36 [35.90–37.82] vs. 35.09 [34.21–36.31] mL/100 g/min, P < 0.0001; CBF in WM: 30.69 [29.84–31.48] vs. 29.30 [28.59–29.99] mL/100 g/min, P < 0.0001; Fig. 4A and B). Furthermore, there were no significant differences in the laterality of CBF in GM and WM between the two time points (Figs. 4C and D).

**Fig. 5:**
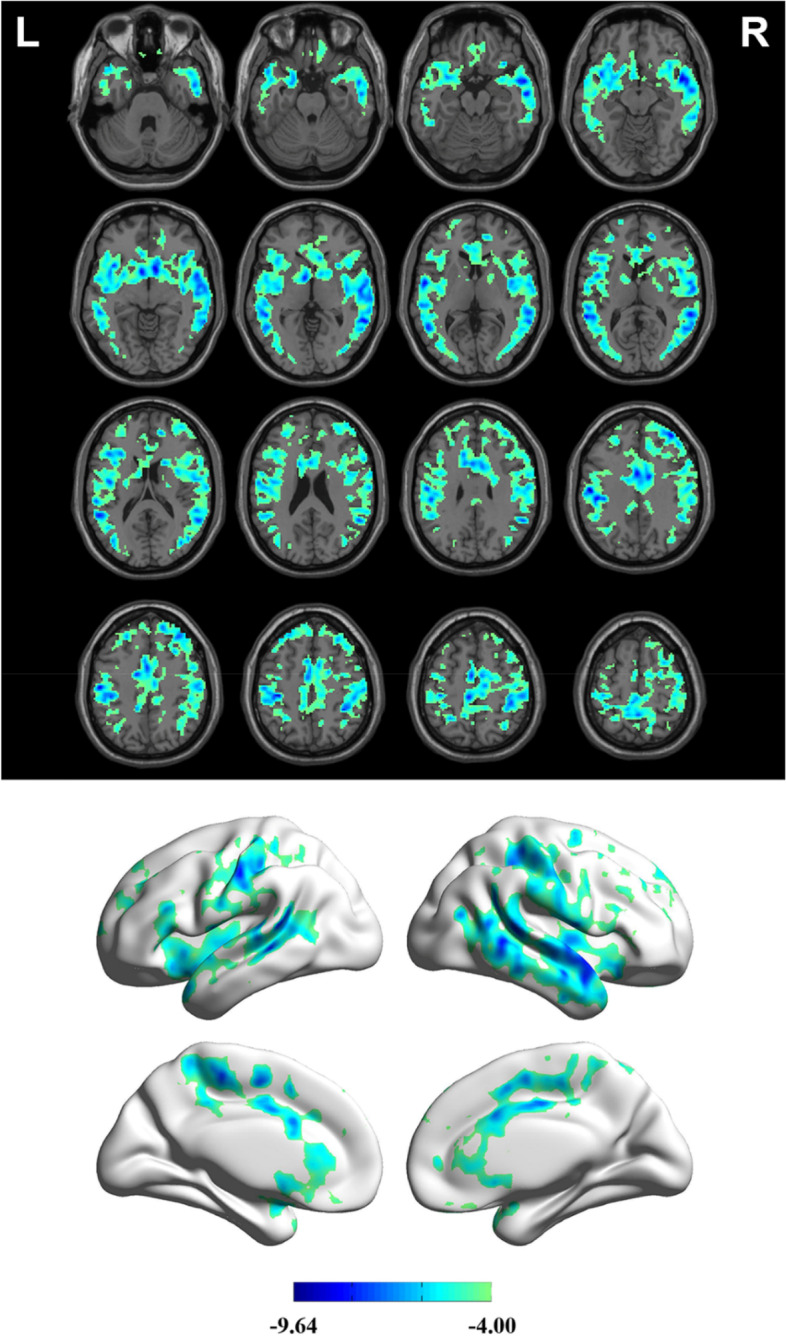
Change in brain blood perfusion after dobutamine infusions compared with the resting state. Voxel-based analysis indicates the brain regions with significant group differences in CBF; the brain regions shown in the figure represent regions with decreased CBF (*P* < 0.001, false discovery rate [FDR] corrected, cluster size > 719). CBF: cerebral blood flow; L: left; R: right

Compared with the CBF in the resting state, the measurements in the stress state showed no significant differences in CBF in the posterior circulation, but there was a significant decrease in the anterior circulation, mainly in the frontal lobe (FDR-corrected; voxel level *P* < 0.001, pixel level *P* < 0.05; cluster size > 719) (Table 2, Fig. 5).

**Table 2:** Brain region with decreased cerebral blood flow after dobutamine infusions

Regions	Cluster size (voxels)	Peak *t* values	MNI coordinates		
			*x*	*y*	*z*
Frontal Lobe	16,225	-9.64	-38	-26	50

*Abbreviations*: *R*, Right, *L*, Left, *MNI*, Montreal Neurological Institute

### Predictors of decrease in CBF

After extracting the CBF in the ROI, we found that the CBF of the 48 subjects decreased from baseline to the stress state. As shown in Table 3, BMI (odds ratio [OR] 5.80, 95% confidence interval [CI] 1.60–21.01, *P* = 0.008), SBP (OR 0.64, 95%CI 0.45–0.92, *P* = 0.014), and BA diameter (OR 11.04, 95%CI 1.05–116.53, *P* = 0.046) were significantly related to the decrease in CBF.

**Table 3:** Multivariate logistic regression analysis results for predicting the impact of dobutamine on brain perfusion

Variable	OR	95% CI	*P*
BMI	5.804	1.596 ~ 21.099	0.008
SBP in resting state	0.644	0.807 ~ 0.955	0.014
BA diameter, mm	11.042	0.000 ~ 0.586	0.046

*SBP* Systolic blood pressure, *BA* Basilar artery, *PCoA* Posterior communicating, *OR* Odds ratio, *CI* Confidence interval

## Discussion

In this study, we used 3D-pcASL to probe the effect of dobutamine on CBF in the entire brain. Two important findings were evident from our data. First, we demonstrated that dobutamine-induced stress decreased CBF in the anterior circulation, mainly in the frontal lobe. Second, we found that the decrease in CBF was associated with a greater BMI, lower baseline SBP, and larger BA diameter.

### Blood pressure and HR responses

Dobutamine is a sympathomimetic agent that increases the myocardial oxygen supply/demand ratio, mainly through positive inotropism. It also has a slight vasodilatory effect on the peripheral vascular resistance, promoting an increase in HR. Increased myocardial contractility and the combination of opposing peripheral vascular actions lead to the overall effect on blood pressure. Previous studies have observed an increase in HR, DBP, and SBP in healthy young men during dobutamine stress echocardiography, which was consistent with our results [30]. Furthermore, a decrease in DBP was observed in patients without known cardiovascular disease, as well as in healthy volunteers, which was not entirely consistent with our results [31, 32]. These studies included patients with coexisting medical conditions that could affect their blood pressure responses to dobutamine stress echocardiography, despite the fact that the patients had no known cardiovascular disease and the volunteers were older than those in our study. Previous studies have shown that older patients develop a blood pressure reduction in response to dobutamine stress echocardiography [32, 33]. These results suggested that the response to dobutamine is age-related, which may be due to age-related decreases in vascular compliance [33, 34].

### CBF response

CBF in all brain regions was within the normal range during dobutamine infusion; however, there was a significant decrease in CBF in the GM and WM, especially in the anterior circulation during dobutamine stress. Consistently, dobutamine was reported to cause a TCDI-estimated decrease in CBF, based on the data from five patients [7]. A previous study suggested that ICA blood flow decreased significantly during heavy exercise, which implies that CBF in the anterior circulation may decrease following a decrease in ICA blood flow [35]. However, dobutamine has been suggested to be associated with a TCDI-estimated increase in CBF in patients with sepsis [11]. The differences between our own and this previous study may be related to the different doses of dobutamine. Another explanation is that patients with circulatory diseases are likely to have disrupted cerebral autoregulation, in which the effects of dobutamine may be stronger. Furthermore, the administration of phenylephrine, a clinically used vasopressor similar to dobutamine, was associated with a trend toward a greater reduction in frontal lobe oxygenation in patients with diabetes [36, 37]. These results support the hypothesis that the frontal lobe is more vulnerable to the effects of vasopressors.

In comparison, the CBF of the posterior circulation, supplied by the vertebral arteries that combine to form the BA, showed no significant change in response to dobutamine. Similarly, the blood flow velocity waveform of the vertebral artery was not altered by dobutamine in a dog model [38]. These results suggest that the posterior circulation is less susceptible than the anterior circulation to the effects of dobutamine. Previous studies have shown that, unlike the ICA, the vascular resistance of the vertebral artery is directly related to the vascular diameter [39, 40]. When the inner diameters of the arteries remain unchanged, the artery presents stable resistance, leading to stable blood flow [41].

Furthermore, there were no significant differences in the laterality of CBF in GM and WM between the two time points, which indicates that the change in CBF in response to dobutamine is symmetric. This may be due to the fact that all volunteers in this study had no cardiovascular or cerebrovascular diseases because the blood flow rate was found to be decreased in the anterior cerebral artery and middle cerebral artery on the ipsilateral (carotid stenosis) side compared with the contralateral side in patients with stroke [42].

### BMI differences

We found that volunteers with higher BMI values during dobutamine stress had a greater decrease in CBF. Higher BMI was found to be associated with lower blood flow velocities in the middle cerebral arteries of patients with type-2 diabetes mellitus, hypertension, and stroke who were divided into three groups based on BMI (normal weight [BMI = 18.5 to 24.9], overweight [BMI 24.9 to 29.9], and obesity [BMI ≥ 30]) [43]. Another study also suggested that a higher BMI correlated with decreased perfusion in all brain regions of patients with Alzheimer’s [44]. These studies suggest that high BMI may adversely affect flow velocity and resistance in the cerebrovascular bed, the cerebrovascular reserve may be limited in human populations with higher BMI and that brain blood flow control may also be impaired. However, clinical studies have shown that the HR response to dobutamine infusion was similar in patients regardless of BMI, in which patients were classified into four groups: < 25 (normal), 25 to 29.9 (overweight), 30 to 39.9 (obese), and ≥ 40 (severely obese) [45]. Therefore, the precise mechanism underlying these BMI differences during dobutamine stress is unclear, and this study’s results may be biased because the sample size of overweight and obese volunteers was small. Further information regarding BMI and CBF is required to explain these responses fully.

### SBP differences

In the present study, the decrease in CBF during dobutamine stress was smaller in volunteers with greater SBP, suggesting that volunteers with greater SBP were less susceptible to CBF decrease during dobutamine stress. In contrast, SBP was negatively related to cortical and hippocampal CBF in subjects without hypertension [46]. However, significant reductions in blood pressure were not associated with changes in CBF in patients with recent stroke [47]. These discrepancies may be caused by intra-subject differences or by the effects of dobutamine. Further studies are needed to address the mechanism underlying these SBP differences in CBF response during dobutamine stress.

### BA diameter

The results of the logistic regression analysis showed that the decrease in CBF was greater in volunteers with larger BA diameters during dobutamine stress. This is likely because an increase in BA diameter leads to a decrease in blood vessel resistance, which further increases blood flow in the BA, according to Poiseuille’s law. The increase in blood flow in the BA may be associated with a decrease in the ICA blood flow, which further results in decreased CBF in the anterior circulation. For example, patients with moyamoya disease, characterized by progressive stenosis or occlusion of the intracranial portion of the ICA and the development of an abnormal vascular network, have a significantly higher flow rate in the BA than do healthy subjects [48].

### Limitations

Our study had several limitations. First, the injected dose of dobutamine was set lower than the routine clinical method to ensure the safety of the subjects. Second, the assessment of cardiac function, such as CO, which is essential for revealing the relationship between the heart and brain under dobutamine injection, was not conducted in the present study, considering that the time taken to measure CO may result in a large infusion amount of dobutamine for the volunteers. Changes in CO have been reported to affect CBF and CO is a vital manifestation after dobutamine injection. Therefore, CO should be considered when exploring the predictors of CBF changes in future studies. Third, there was a sex imbalance in the study, which limited the ability to explore sex differences in dobutamine-induced effects. Fourth, arterial transit time (ATT) and cerebral blood volume (CBV) could not be provided because the single post-labeling delay (PLD) was adopted in this study. In the future, ATT and CBV should be considered in studies on CBF changes by using multi-PLDs PCASL, by which CBF and ATT can be estimated, reducing the bias caused by unknown ATT and also providing extra, potentially clinically useful, information [49, 50]. Fifth, the sample size is small, especially the proportion of volunteers who are overweight or obese, which may lead to unrepresentative results and possible bias, such as the relationship between BMI and CBF.

## Conclusions

In conclusion, dobutamine-induced stress decreased CBF in the anterior circulation, mainly in the frontal lobe. The predictors of a hypotensive response on multivariate analysis were a higher BMI, lower baseline SBP, and greater BA diameter. These findings suggest that anterior circulation is highly susceptible to sudden changes in cardiac function. However, the exact mechanisms and causative relationships remain unclear. Therefore, functional changes in the anterior circulation of patients need to be monitored during clinical dobutamine use.

## Data Availability

The data used to support the findings of this study are available from the corresponding authors upon request.

## Abbreviations

CBF: Cerebral blood flow
TCDI: Transcranial color Doppler imaging
ASL: Arterial spin labeling
3D-pcASL: Three-dimensional pseudocontinuous ASL
ICA: Internal carotid artery
BA: Basilar artery
ROI: Regions of interest
SBP: Systolic blood pressure
DBP: Diastolic blood pressure
ATT: Arterial transit time
CBV: Cerebral blood volume
PLD: Post-labeling delay
